# Effect of Exercise Combined with Natural Stimulation on Korean College Students’ Concentration and Positive Psychological Capital: A Pilot Study

**DOI:** 10.3390/healthcare10040673

**Published:** 2022-04-02

**Authors:** Kyudong Lee, Hwanseong Bae, Seyong Jang

**Affiliations:** 1 Martial Arts and Physical Education Center, Korean National Police University, Asan 31539, Korea; lkd13579@police.go.kr; 2 Department of Taekwondo, College of Arts and Physical Education, Gachon University, Seongnam 13120, Korea

**Keywords:** college students, concentration, exercise, healing, natural environment, natural stimulation, perceived restorativeness, positive psychological capital

## Abstract

This study aimed to investigate the effects of short-term exercise, within the natural environment or by applying similar visual stimulation, on concentration and positive psychological capital among Korean college students. Participants were 175 male college students—selected by non-probabilistic sampling—from the Korean National Police University in Asan-si, Republic of Korea, in March 2021. Participants were divided into three condition groups: the natural environmental exposure with outdoor exercise (*n* = 57), visual stimulation with indoor exercise (*n* = 58), and indoor exercise (control group; *n* = 60). The variables measured were concentration and positive psychological capital. Pre- and post-exercise data differences were analyzed using two-way (3 × 2) analysis of variance and Pearson’s correlation analysis, and statistical significance was set at 0.05. The results revealed a significant main effect on concentration, with lower scores post-intervention indicating positive changes in all three groups. In addition, the scores for positive psychological capital sub-factors (self-efficacy, optimism, and hope), in the groups with the natural environmental exposure with outdoor exercise and visual stimulation with indoor exercise conditions, reflected higher positive change than the indoor exercise group (*p* < 0.05). Furthermore, the Bonferroni post hoc test on this interaction effect revealed that the participant scores for the natural environmental exposure with outdoor exercise and visual stimulation with indoor exercise groups were positive after the exercise (*p* < 0.05). However, there was no interaction effect for the ego-resilience subscale (*p* > 0.05). Therefore, participating in short-term exercise while being exposed to a natural environment with healing characteristics or providing visual stimulation of a similar natural environment was found to positively impact the Korean college students’ concentration and positive psychological capital’s self-efficacy, optimism, and hope. Moreover, this particular intervention only affects subjective measures of well-being while not particularly influencing objective measures, such as cognitive functioning. We recommend implementing similar visual stimulation with indoor exercise for the current generation during the COVID-19 pandemic.

## 1. Introduction

Capitalism and its constant growth act as a driving force in providing material abundance and a safe and comfortable living environment. However, the current generation of people include college students, who are forced to devote a lot of their daily lives to surviving in an extremely competitive social atmosphere by working in their company or studying at their college in indoor spaces [1,2,3]. Moreover, a major proportion of the current generation’s indoor life is dedicated to office work or college lectures at desks with little movement; thus, they are directly exposed to health threats imposed by a lack of physical activity or exercise [3]. Several studies focused on college students [1,4] and various age groups [5] are being published to verify the effectiveness of physical activity or exercise for promoting health in the current generation, and they report that participation in exercise positively affects physical and mental health while relieving stress. Additionally, scientific evidence, suggesting that exposure to the natural environment positively affects current generation’s health, has recently gained significant attention in the relevant fields [6,7,8,9,10,11].

Existing studies on the positive effect of exposure to the natural environment report that patients in the hospital room, who can see plants and other natural views outside through the window, exhibit positive results for headaches, pain, complaints, and recovery period, with reduced physical pain and mental stress than those who do not see these views [1,4,6,7]. Moreover, participants reported lowered pulse rate and blood pressure and improved stability of the autonomic nervous system while observing the natural environment with plants than in the urban jungle [12]. In addition, studies using an electroencephalogram revealed increased alpha waves as the green area of the visually visible plant increased [13,14]. Based on the fact that the time it takes to solve a given problem is reduced compared to the group that did not see plants, it is predicted that the visual stimulation of plants is one of the factors that increase the activity of the parts responsible for human thinking and memory [15]. With the growing body of literature on the positive effects of exposure to the natural environment for the current generation, a new theme has begun to emerge around the keywords natural environment and exercise [3,4,5,16].

Some relatively recent research has examined the improved effectiveness of combining exercise with the natural environment and obtained remarkable results by verifying the effectiveness of exercise in natural environments such as forests and parks [3,4,5,16]. Previous studies have demonstrated the positive effect of groups participating in exercise within a natural environment (e.g., walking on forest paths) on participants’ concentration, blood pressure and tension, anxiety, mood conditions, and perceived exercise intensity [1,4,5,16,17]. Interestingly, these studies reported that when exercise is performed in the natural environment rather than indoors, it has more psychological and physiological positive effects. These results show that oxygen plays an essential role for efficient energy generation and consumption, oxygen intake increases via increased exercise intensity, and the place (e.g., forest or mountain paths) where exercise is performed has a positive effect on psychological and physiological variables by purified air pollutants via the natural environment [18,19].

The concentration restoration theory provides a theoretical basis for explaining the effects of exercise in the natural environment [20], and it proposes the healing characteristics of the natural environment. According to this theory, there is a need to implement methods to restore concentration, similar to methods applied for restoring depleted physical strength, because human concentration is easily depleted, causing mental fatigue, impulsiveness, and frequent mistakes in performance [21]. The consumed concentration is particularly recovered through visual stimulation or activities related to environments with healing characteristics, which are typically inherent to the natural environment [20,21,22]. Here, the healing characteristics of the natural environment imply those characteristics that restore human psychological and physical conditions to its original state and promote health. Furthermore, it has been reported that the positive healing effect of natural scenery is consistent across diverse socioeconomic characteristics, such as race or culture [12,23]. Thus, it is crucial and meaningful to examine the positive effect of exercise through various forms of exposure to the natural environment for enhancing the current generation’s mental and physical health. However, it is practically difficult for the current generation to survive in a fiercely competitive society while continuing to exercise in the long term within the natural environment, as they spend over 80% of their daily lives within indoor spaces. Furthermore, environmental threats, such as fine dust and the COVID-19 pandemic, are limiting outdoor physical activities and raising negative risks, such as anxiety regarding outdoor activities [24].

Therefore, research is required to transfer and increase the positive effects of outdoor exercise to indoor exercise and to apply the healing characteristics of the natural environment to indoor places, where a majority of the current generation exercises. However, existing research on this topic is insufficient. Therefore, this study aimed to confirm the effects of short-term exercise using visual stimulation or the natural environment on the concentration and positive psychological capital of Korean college students. Thus, it can be helpful in providing basic information to improve the quality and effects of exercise.

## 2. Methods

### 2.1. Participants

Participants were 175 male college students, assessed for eligibility (*n* = 175). Participants were selected through non-probabilistic sampling—from the Korean National Police University in Asan-si, Republic of Korea in March 2021—and no participants were excluded, as all college students satisfied the inclusion criteria and provided consent to participate. These participants were randomly divided into three groups: natural environmental exposure with outdoor exercise (*n* = 57), visual stimulation with indoor exercise (*n* = 58), and indoor exercise (control group; *n* = 60). The sample size was determined by performing a two-way (3 × 2) analysis of variance using the G*Power software (version 3.1.9.7, Heinrich-Heine-University, Germany) to obtain the optimal power (1-beta) and significance level (*p* < 0.05). This analysis prompted a suitable sample size of 63 participants to obtain a power and effect size (d) of 0.80 and 0.50, respectively [15]. Nevertheless, we recruited 175 male participants to increase the reliability of the study, with as many participants as possible from the Korean National Police University. Moreover, the current study’s participants were students at a professional university called the Korean National Police University, and the proportion of female students at this school was very small (less than 3%). Thus, females were not included in the study to avoid the assumption of equal variance and the presence of extreme values.

The inclusion criteria for participant recruitment in this study were: (1) those who were males aged 20–23 years old, (2) those who studied at the Korean National Police University, and (3) those who provided consent to participate. Participants who did not satisfy any of these criteria were excluded; however, all eligible participants in this study satisfied the criteria and were recruited. We obtained written informed consent from participants prior to their voluntary participation. The study was conducted in accordance with the principles outlined in the Declaration of Helsinki and approved by the Institutional Review Board of Gachon University (1044396-202103-HR-060-01). Table 1 presents the participants’ characteristics.

### 2.2. Instruments

A cognitive function test (Trail-Making Test Part 2) was used to measure participants’ concentration levels. This test is popularly used to measure concentration and was initially used for examination within the field of neuropsychology [25]. Participants had to use a pen and pull a line between points without lifting their hand in the following sequence—1–A–2–B–3–C; the time taken to complete the exercise was measured. This evaluation method has been used since 1958 to measure participants’ test completion time without mistakes and their promptness to modify their mistakes [17,25].

The positive psychological capital test was developed by Luthans et al. [26]. This test comprises four subscales (self-efficacy, optimism, hope, and ego-resilience) and includes a total of 18 items rated on a 5-point Likert scale, ranging from 1 (*not at all*) to 5 (*very much so*). Examples of an item for each subscale are as follows: “I can successfully overcome difficult things,” “I am always optimistic about my future,” “Now I am moving vigorously toward my goal,” and “I tend to recover quickly even if I go through difficult things.” Further details on all items are described in Luthans et al. [26]. Table 2 and Table 3 present the psychometric validation results as measured twice before and after the experiment. The central limit theorem establishes that a sample with more than 30 measurements will report a normal-like distribution and will be reliable [27]. Considering our current study’s participants (*n* = 175), we believed that the sample size was adequate.

### 2.3. Experimental Procedure

#### 2.3.1. Exercise Routine

The current study exercise routine implemented the Harvard step exercise method based on the book of *Advanced Fitness Assessment and Exercise Prescription* [28] to increase participant efficiency and enable consistency in the exercise intensity of multiple participants simultaneously. Moreover, a 43 cm-high step box was used for the Harvard step to ensure consistency across all participant groups, and the exercise routine spanned a total of 40 min (10-min exercise, 5-min break, 10-min exercise, 5-min break, and 10-min exercise). Since exercise was conducted in a single session, we defined this routine as “short-term exercise”. All three participant groups performed the same exercise routine, and consistency in exercise intensity was ensured by conducting a test of subjective exercise intensity (Rating of perceived exertion) created by Borg and Noble [29]. This test evaluates the degree of fatigue based on the participants’ heart rate and presents an index on a 15-stage range from 6 (lowest intensity (during rest)) to 20 (maximum intensity). The test was self-administered after the completion of the exercise routine and was used to measure the degree of awareness of exercise intensity. Participants demonstrated an average of 11.67 points on the rating of perceived exertion during exercise.

#### 2.3.2. Experimental Treatments

**Figure 1:**
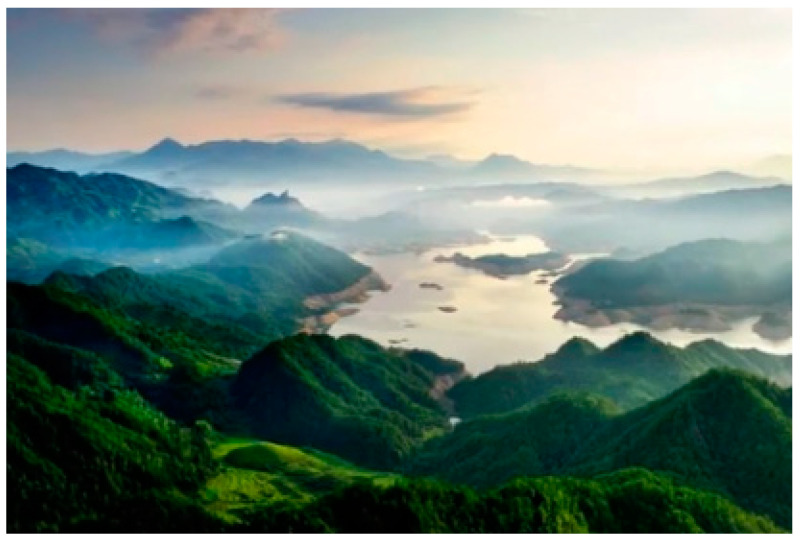
Illustration of a forest.

During the exercise, participants in the natural environmental exposure with outdoor exercise group were exposed to a real forest. Similarly, participants in the visual stimulation with indoor exercise group were presented with an image based on the natural environment with healing characteristics (Figure 1). Moreover, for this group, the natural environment with healing characteristics was completed by repeatedly showing several photos with well-expressed natural scenery and selecting photos closest to the research purpose. In this study, photos containing natural scenery were selected by distributing them to 175 participants and observing their feelings toward the photos. Subsequently, we produced and presented the selected photos with large outdoors, as pre-timed video slides on Microsoft^®^ Office PowerPoint 2019 (Microsoft Corporation, Redmond, WA, USA), to display the finished images on a wide screen, making it easier for participants to perform the experimental task efficiently while engaging with visual stimuli. The indoor exercise group (control group) exercised indoors with no environmental treatments.

### 2.4. Statistical Analyses

Data were analyzed using SPSS 23.0 and AMOS 23.0 software (IBM Corp., Armonk, NY, USA). First, a descriptive statistical analysis was conducted to determine study participants’ general characteristics. Second, the positive psychological capital test was psychometrically validated by performing confirmatory factor analysis and reliability analysis. Third, a two-way (3 × 2) analysis of variance and Pearson’s correlation analysis were performed to examine the difference between the concentration levels and positive psychological capital before and after the exercise routine between the three participant groups. Furthermore, a Bonferroni post hoc test was performed to determine if the interaction effect was statistically significant. Statistical significance was set at *p* < 0.05.

## 3. Results

### 3.1. Concentration

Table 4 presents the results of the analysis. The correlations between pre-and post-exercise scores were 0.543 (*p* < 0.001) and 0.356 (*p* < 0.01) for participants in the natural environmental exposure with outdoor exercise and visual stimulation with indoor exercise groups, respectively. It was found that the time taken on the concentration task trial was significantly lower, indicating improved concentration and a significant main effect in the post-test than the pre-test (*p* < 0.001).

### 3.2. Positive Psychological Capital

Table 5 presents the results of the analysis. The results revealed a significant interaction effect of group × trial on self-efficacy, optimism, and hope (*p* < 0.05). Furthermore, the Bonferroni post hoc test on the interaction effect reported that the participant scores for the natural environmental exposure with outdoor exercise and visual stimulation with indoor exercise groups were positive after the exercise (*p* < 0.05). However, there was no interaction effect for the subscale of ego-resilience.

## 4. Discussion

This study aimed to present basic data to improve the effectiveness of exercise by examining the impact of short-term exercise on concentration and positive psychological capital based on theoretical evidence for the positive effect of visual stimulation of the natural environment.

With the vast expanse of information that modern society is exposed to, information processing ability becomes an essential element to effectively solve various problems. Attention is the first step in the human information processing process, and any defects in attention, concentration, and information processing create difficulties in problem solving; thus, attention and concentration ability is of utmost importance for processing information. The analyses of the differences pre- and post-exercise revealed that in all groups, concentration improved significantly (faster task completion time) after exercise. This result is consistent with previous study findings [30,31], which reported a strong positive correlation between cognitive function and exercise before and after performing aerobic exercise [30,31]. In addition, short-term exercise participation in this study helped improve participants’ concentration. This finding was supported by a study indicating improvements in students’ cognitive functions (e.g., short-term memory or selective attention) and concentration through participation in exercise [32]. The current study’s implications are significant due to the impact of short-term exercise on concentration as well as cognitive function, including intellectual abilities such as processing and storing information obtained from various sources, memory, judgment, reasoning, and calculation [14,30,31,32]. This decline in time needed to complete the task resulting in better performance observed in cognitive function is considered to be a very meaningful result of this study, indicating that short-term exercise helps improve concentration.

The results revealed a statistically significant interaction effect between the positive psychological capital subscales of self-efficacy, optimism, and hope. Self-efficacy is a concept introduced by Bandura [33] and is defined as judgment or belief in one’s ability to successfully perform the given tasks. Individuals with higher self-efficacy have been found to successfully perform a given task regardless of their ability [33]. These results propose a meaningful approach to improve college students’ self-efficacy from various perspectives; the current study findings show that the participant scores for the natural environmental exposure with outdoor exercise and the visual stimulation with indoor exercise groups changed positively after exercising.

Optimism refers to a positive outlook with the belief that life will continue to improve despite the various difficulties and setbacks experienced [26]. Scheier and Carver [34] published a study reporting that people with high optimism levels tend to believe more good things will happen than bad things, and they tend to demonstrate an active behavioral attitude consistent with their positive expectations. Seligman [35] particularly argued that by applying a method that does not pinpoint one’s faults when a negative event occurs, one can avoid being engulfed in negative emotions, such as guilt or depression, and pessimists can learn optimism. When this optimism is mastered, the attributes to be maintained are strongly formed. Thus, it is argued that an individual’s life can be positively changed. Optimism can be learnt by controlling negative emotions and strengthening positive tendencies. In addition, despite the common tendency for humans to experience negative emotions, accompanied by depression and anxiety, upon encountering unhappy events, people with high optimism suffer less psychologically in such situations [34,35]. Such highly optimistic individuals use positive emotions to adapt to the situation more easily; thereby, exercising in an environment with therapeutic qualities improves participants’ optimism [1,35]. Optimism is a very important trait for individual growth from a developmental perspective, while, simultaneously, it demonstrates a positive effect on college students’ life changes as they face various stressors in line with the changes in their social environment [1,35].

Hope generally refers to the expectation of a bright future or that good things will happen, and it indicates positive motivation according to the interaction between a plan to achieve one’s goal and a strong will [36]. Peterson et al. [37] regard hope as a positive response to a situation in which one’s actions toward the future are constrained. Luthans and Youssef [26] found that people with higher hope levels exhibit a higher probability of success than those with lower hope levels. This finding can be partially explained by Snyder’s [3] findings, which indicate that hope maintained a positive emotional state that focuses on success rather than failure and recognizes a high possibility of success. In particular, people belonging to groups with relatively higher levels of hope perceive their social competence as sufficient, invest a relatively large amount of time to socialize, take initiative in interpersonal relationships, and feel less lonely [36,37]. In this study, the groups provided with the natural environment and visual stimulus reported positive influences on their scores than the control group. It implies a very meaningful finding suggesting that the award is based on smooth interpersonal relationships.

Contrary to self-efficacy, optimism, and hope, there was no significant difference in the interaction effect between groups on ego-resilience. Typically, ego-resilience is related to commitment and job satisfaction, and it is commonly viewed as independent from hope or optimism, which are focused on regarding the active aspect of humans [26]. Thus, resilience can be interpreted as a constructive concept that differs from the aforementioned three components, in that it is explained as a direct or indirect response to the difficulties currently experienced. Nevertheless, resilience can further solidify an individual’s perceived positive emotional level as it interacts with self-efficacy, optimism, and hope—the components of positive psychological capital. However, the results of this study revealed a positive effect of exercise on ego-resilience as the scores of all groups improved for ego resilience after exercise.

In accordance with the findings suggesting that those exposed to high-quality natural landscapes with healing elements report higher psychological stability than those exposed to regular landscapes [38,39], we examined the differences in positive psychological capital through its sub-factors. The results revealed a significant interaction effect between the sub-factors of efficacy, optimism, and hope, indicating higher positive changes in the scores of the groups in the natural environment and visual stimulation exercise conditions than those in the indoor exercise group. These results lend support to previous studies that theorize that the visual stimulation of the natural environment with healing properties demonstrates a similar effect to that of the actual exposure to the natural environment [12,13,14,15]. In particular, the positive change in the scores of the natural environment and visual stimulus exercise condition groups indicates that the importance of the visual environment among the physical components of the environment with healing properties has a positive effect on human physiological, psychological, and behavioral responses [40].

In addition, a physical environment with healing properties enables human psychological, mental, and physical fatigue to recover to a stable state. Based on a previous study reporting the partial mediating role of psychological capital, the logical validity of the current study results may be improved. Furthermore, it also supports the belief that positive psychological capital will continue to improve with a positive outlook despite various difficulties and setbacks experienced in human life [36]. The positive changes in hope—the expectation of what will happen or a bright prospect—and the positive motivation that comes from the interaction between a plan to achieve one’s goal and strong will are considered to be remarkable findings [41]

This study has several limitations due to the short-term exercise intervention implemented in this study. First, due to the short-term nature of the intervention, participants recruited in this study were not entirely representative of the general population. Second, only two psychological factors—concentration and positive psychological capital—were evaluated in this study. Third, the Harvard step exercise method used in this study may not be entirely appropriate for practice in the natural environment. Therefore, in subsequent studies, it will be necessary to apply suitable and diverse short-term exercises within the natural environment. These studies will help us obtain scientific and objective information to improve participants’ performance. Fourth, the measurement tool for concentration may have been biased due to a learning effect among the study participants. Therefore, future studies should control this learning effect by applying various strategies (e.g., randomizing the order). Fifth, several uncontrollable factors in the natural environment, such as temperature, humidity, sound, light, environmental changes, and so on, were not considered in this study. In addition, multidisciplinary studies should be promoted to analyze various factors, including physiological factors, besides the psychological factors in this study. Sixth, in this study, we did not evaluate the accumulation of fatigue in each group; however, measuring fatigue may be helpful in future studies. Seventh, in this professional university, the proportion of female students at this school was very small (less than 3%). Thus, females were not included in the study to avoid the assumption of equal variance and the presence of extreme values. This may have limited the generalizability of the study findings. Therefore, well-designed studies including both males and females are warranted in the future. Lastly, there is a need to recruit participants of various age groups in large numbers, participants without extra activities in the office, and to include the convalescent hospital patients with subdivided time periods in further research.

## 5. Conclusions

This study concluded that short-term exercise performed within a natural environment with healing characteristics or alongside visual stimulation of a similar natural environment positively affected Korean college students’ concentration and their self-efficacy, optimism, and hope of positive psychological capital. Moreover, this particular intervention only affected subjective measures of well-being, while no particular influences were imposed on objective measures, such as cognitive functioning. We recommend the implementation of visual stimulation of a natural environment with indoor exercise to help the current generation during the COVID-19 pandemic.

## Figures and Tables

**Table 1:** Participant characteristics (n = 175).

Variables	Natural Environmental Exposure with Outdoor Exercise Group (n = 57)	Visual Stimulation with Indoor Exercise Group (n = 58)	Indoor Exercise Group (Control Group) (n = 60)
Age (in years)	22.25 ± 0.87	21.26 ± 0.87	20.60 ± 0.88
Height (cm)	174.49 ± 5.04	174.71 ± 4.11	176.13 ± 5.65
Weight (kg)	71.76 ± 9.98	70.65 ± 8.25	70.44 ± 9.26
Body mass index (kg/m^2^)	23.54 ± 2.91	23.12 ± 2.34	22.86 ± 2.82

Values are presented as mean ± standard deviation.

**Table 2:** Construct validity results via confirmatory factor analysis for the positive psychological capital test.

Variables	$\chi^{2}(\mathrm{df}),\;p-\mathrm{Value}$	Q-Value	Comparative Fit Index	Tucker–Lewis Index	Root Mean Square Error of Approximation
Pre-experimental	236.281 (129), *p* < 0.001	1.832	0.948	0.938	0.069
Post- experimental	288.686 (129), *p* < 0.001	2.255	0.927	0.913	0.077
Criterion	*p* > 0.05	<3.000	>0.900	>0.900	<0.080
Decision	Reject	Accept	Accept	Accept	Accept

**Table 3:** Reliability results via Cronbach’s alpha for the positive psychological capital test.

Variables	Subscales	Average Variance Extracted	Construct Reliability	Cronbach’s Alpha
Pre-experimental	Self-efficacy	0.789	0.949	0.909
	Optimism	0.645	0.899	0.858
	Hope	0.726	0.929	0.881
	Ego-resilience	0.768	0.909	0.900
Post-experimental	Self-efficacy	0.872	0.972	0.940
	Optimism	0.614	0.887	0.796
	Hope	0.747	0.937	0.886
	Ego-resilience	0.792	0.919	0.900

**Table 4:** Concentration pre- and post-exercise among the three participant groups.

Groups		*n*	Pre-Exercise (s)		Post-Exercise (s)		Pre-Exercise and Post-Exercise Correlation (r)
			Mean	Standard Deviation	Mean	Standard Deviation	
Natural environmental exposure with outdoor exercise		57	49.47	15.91	34.62	11.00	0.543 ***
Visual stimulation with indoor exercise		58	50.20	11.12	36.55	11.43	0.356 **
Indoor exercise group		60	51.32	15.72	37.94	13.69	0.121
Total		175	50.35	14.37	36.40	12.13	0.359 ***
Source	Sum of squares	*df*	Mean square	*F*	*p*	$\eta^{2}$	Post hoc (Bonferroni)
Group	390.374	2	195.187	0.814	0.445	0.009	-
Trial	17,041.366	1	17,041.366	147.814	<0.001	0.462	Pre > Post ***
Group X Trial	35.056	2	17.528	0.152	0.859	0.002	-

** *p* < 0.01, *** *p* < 0.001; tested by mixed-model two-way (3 × 2) analysis of variance and Pearson correlation analysis; df: degree of freedom, group: natural environmental exposure with outdoor exercise group, visual stimulation with indoor exercise group, and indoor exercise group; trial: pre-experimental and post-experimental.

**Table 5:** Positive psychological capital pre- and post-exercise among the three participant groups.

Groups	*n*	Self-Efficacy (Score)			Optimism (Score)			Hope (Score)			Ego-Resilience (Score)		
		Pre	Post	r	Pre	Post	r	Pre	Post	r	Pre	Post	r
G1	57	3.79 ± 0.68	3.97 ± 0.71	0.781 ***	3.71 ± 0.71	3.99 ± 0.63	0.581 ***	3.76 ± 0.66	3.92 ± 0.66	0.708 ***	3.65 ± 0.91	3.86 ± 0.86	0.688 ***
G2	58	3.75 ± 0.66	3.99 ± 0.62	0.734 ***	3.74 ± 0.75	3.99 ± 0.42	0.504 ***	3.81 ± 0.80	3.96 ± 0.69	0.891 ***	3.68 ± 0.90	3.91 ± 0.81	0.849 ***
G3	60	4.06 ± 0.51	4.05 ± 0.50	0.828 ***	3.73 ± 0.58	3.64 ± 0.47	0.307 ***	3.83 ± 0.49	3.82 ± 0.49	0.786 ***	3.68 ± 075	3.89 ± 0.67	0.800 ***
Total	175	3.87 ± 0.63	4.00 ± 0.61	0.765 ***	3.73 ± 0.68	3.87 ± 0.54	0.448 ***	3.80 ± 0.66	3.90 ± 0.62	0.797 ***	3.67 ± 0.85	3.89 ± 0.78	0.775 ***
Variables	Source				Sum of squares	*df*	Mean square	*F*	*p*	$\eta^{2}$	Post hoc (Bonferroni)		
Self-efficacy	Group				2.471	2	1.236	1.838	0.162	0.021	-		
	Trial				1.608	1	1.608	18.792	< 0.001	0.098	Pre < Post ***		
	Group X Trial				1.019	2	0.510	5.957	0.003	0.065	G1: Pre < Post **/G2: Pre < Post ***		
Optimism	Group				2.327	2	1.164	2.199	0.114	0.025	-		
	Trial				1.787	1	1.787	9.023	0.003	0.050	Pre < Post **		
	Group X Trial				2.557	2	1.278	6.455	0.002	0.070	G1: Pre < Post **/G2: Pre < Post **		
Hope	Group				0.224	2	0.112	0.152	0.859	0.002	-		
	Trial				0.873	1	0.873	10.804	0.001	0.059	Pre < Post **		
	Group X Trial				0.570	2	0.285	3.526	0.032	0.039	G1: Pre < Post **/G2: Pre < Post *		
Ego-resilience	Group				0.093	2	0.047	0.039	0.962	0.000	-		
	Trial				4.125	1	4.125	26.965	<0.001	0.136	Pre < Post **		
	Group X Trial				0.007	2	0.004	0.023	0.977	0.000	-		

Data are expressed as mean ± standard deviation. G1; natural environmental exposure with outdoor exercise, G2; visual stimulation with indoor exercise, G3; indoor exercise group. * *p* < 0.05, ** *p* < 0.01, *** *p* < 0.001; tested by two-way (3 × 2) analysis of variance and Pearson correlation analysis.

## Data Availability

The data presented in this study are available on request to the authors.
